# *Polygonum cognatum* Powder as a Functional Ingredient: Improving Nutritional Quality and Reducing Glycemic Index of Gluten-free and Wheat-based Crackers

**DOI:** 10.1007/s11130-026-01518-9

**Published:** 2026-05-21

**Authors:** Tekmile Cankurtaran-Kömürcü, Nermin Bilgiçli

**Affiliations:** Department of Food Engineering, Engineering Faculty, Necmettin Erbakan University, Köyceğiz Campus, Konya, 42050 Turkey

**Keywords:** *Polygonum cognatum*, Functional food, Antioxidant activity, Glycemic index, Crackers

## Abstract

*Polygonum cognatum* is an underutilized edible plant rich in dietary fiber, minerals, and bioactive compounds with potential applications in functional foods. This study evaluated the effects of *Polygonum cognatum* powder (PCP) incorporation (0–15%) on the nutritional, bioactive, technological, sensory, and glycemic properties of gluten-free and wheat-based crackers. PCP addition significantly (*p* < 0.05) increased protein, total dietary fiber, phenolic content, antioxidant capacity, and mineral composition (Ca, Fe, Mg, K, and P), while reducing phytic acid levels in both formulations. Antioxidant activity (DPPH, FRAP, and CUPRAC) and total phenolic content increased markedly in a dose-dependent manner, indicating the high bioactive potential of PCP. In vitro starch digestion analysis showed that PCP incorporation effectively reduced the estimated glycemic index (eGI), with values decreasing from 92.45 to 57.98 in wheat-based crackers and from 96.57 to 60.12 in gluten-free crackers. Gluten-free samples consistently exhibited slightly higher eGI values, likely due to the higher digestibility of starch-based matrices. PCP addition increased spread ratio and hardness, Spread ratio and hardness increased above 3% PCP substitution level. Sensory evaluation indicated that 3–6% substitution provided optimal acceptability, whereas higher levels (≥ 12%) negatively affected color, taste, and crispness. Overall, PCP is a promising functional ingredient for developing nutritionally enhanced and lower-glycemic bakery products.

**Supplementary Information:**

The online version contains supplementary material available at 10.1007/s11130-026-01518-9.

## Introduction

*Polygonum cognatum* Meissn. is a wild edible plant widely distributed across Türkiye, the Caucasus, the Middle East, and parts of Central Asia, where it has traditionally been consumed as a seasonal leafy vegetable. The young shoots of the plant are typically harvested in spring and used in various local dishes [1]. In addition to its culinary use, *P. cognatum* has been employed in traditional medicine for the treatment of several ailments, including rheumatic disorders, gastrointestinal discomfort, anemia, and skin-related conditions, and is also reported to exhibit diuretic and antidiabetic effects [2]. Despite its long-standing ethnobotanical relevance, *P. cognatum* remains insufficiently explored in terms of its nutritional composition and functional potential compared to other commonly consumed leafy vegetables. Previous studies have primarily focused on specific properties such as antioxidant activity and antimicrobial effects [3], the impact of processing conditions on color characteristics [2], and changes in bioactive compounds during storage [4]. However, comprehensive investigations addressing both the nutritional quality and functional applications of *P. cognatum*, particularly in processed food systems, are still limited.

In recent years, there has been increasing interest in the development of functional foods enriched with plant-based ingredients, particularly in cereal-based products such as crackers and snacks. The incorporation of plant-derived powders into bakery formulations has been reported to enhance dietary fiber content, phenolic compounds, antioxidant capacity, and mineral composition [5, 6]. Moreover, such enrichment strategies may contribute to improved health-related properties, including modulation of glycemic response, which is of particular importance in the prevention of metabolic disorders [7]. These approaches are especially relevant for gluten-free products, which are often characterized by lower nutritional quality and higher glycemic index compared to wheat-based counterparts [8].

In this context, the incorporation of underutilized plant materials such as *P. cognatum* into bakery products represents a promising strategy to improve both nutritional value and functional performance. Therefore, the aim of this study was to evaluate the effects of PCP incorporation on the nutritional, bioactive, technological, sensory, and glycemic properties of both gluten-free and wheat-based cracker formulations. The findings of this study are expected to contribute to the valorization of this traditional plant and support its use in the development of functional cereal-based foods.

## Materials and Methods

The materials and detailed procedures are described in the Supplementary Material.

## Results and Discussion

### Color Properties of Crackers Samples

The incorporation of PCP significantly (*p* < 0.05) influenced the color properties of both gluten-free and wheat-based crackers (Table 1). The most pronounced effect was the reduction in L*, which decreased from 78.61 (gluten-free) and 81.64 (wheat-based) in the control samples to approximately 48 at 15% PCP, indicating substantial darkening. This darkening may also be associated with the oxidation and polymerization of phenolic compounds during baking, together with the contribution of chlorophyll and other natural pigments present in PCP. In addition, non-enzymatic browning reactions, including Maillard reactions, may have contributed to color development during baking; however, in plant-based systems rich in natural pigments and phenolic compounds, these reactions alone may not fully account for the pronounced color changes observed in PCP-enriched crackers [9, 10]. Similar reductions in L* values have been reported in bakery products enriched with plant-based powders, which are generally associated with the presence of chlorophylls and phenolic compounds as well as non-enzymatic browning reactions occurring during baking [11]. Increasing PCP also shifted a* values toward more negative numbers, enhancing greenness, with the lowest values recorded at 15% substitution (−10.18 in gluten-free and − 9.82 in wheat-based crackers), consistent with the high chlorophyll content of PCP. This trend agrees with previous findings indicating that incorporation of leafy plant materials leads to increased greenness in cereal-based products due to their high chlorophyll content [12]. Yellowness declined with increasing PCP levels in gluten-free crackers. In wheat-based crackers, the addition of PCP resulted in a reduction in yellowness, but this reduction was only significant (*p* < 0.05) at addition levels above 9%. Similar decreases in b* values have been reported in bakery systems enriched with fiber- and phenolic-rich ingredients, which affect light absorption and color perception [13]. Hue values increased (from 97.58 to 111.28 in wheat-based crackers), confirming the dominance of green tones, while SI values decreased in gluten-free formulation reflecting reduced color vividness. When gluten-free and wheat-based crackers are compared in terms of average color values, only the L* value is significantly (*p* < 0.05) higher in wheat-based crackers. No significant differences were found between other color values.

**Table 1:** Color properties of gluten-free and regular cracker samples^1^

Type of cracker	PCP^2^ ratios (%)	L*	a*	b*	Hue	SI
Gluten-free	0	78.61 ± 0.32a	−5.62 ± 0.71a	37.66 ± 1.29a	98.49 ± 1.37d	38.08 ± 1.17a
	3	62.81 ± 0.16b	−6.45 ± 0.32ab	34.42 ± 0.51b	100.61 ± 0.66 cd	35.02 ± 0.44b
	6	60.28 ± 0.24c	−7.00 ± 0.09b	33.86 ± 0.72b	101.68 ± 0.10c	34.57 ± 0.72b
	9	53.88 ± 0.32d	−8.65 ± 0.01c	30.67 ± 0.88c	105.75 ± 0.44b	31.86 ± 0.85c
	12	50.54 ± 0.18e	−9.04 ± 0.40c	28.83 ± 0.86 cd	107.44 ± 1.22b	30.22 ± 0.70 cd
	15	48.43 ± 0.17f	−10.18 ± 0.04d	27.05 ± 0.45d	110.66 ± 0.35a	28.90 ± 0.41d
Wheat-based	0	81.64 ± 0.21a	−4.63 ± 0.21a	34.84 ± 1.12a	97.58 ± 0.58e	35.14 ± 1.08a
	3	70.49 ± 1.29b	−6.42 ± 1.29ab	33.60 ± 0.29a	100.82 ± 2.06d	34.21 ± 0.52a
	6	60.50 ± 0.93c	−8.29 ± 0.93bc	33.04 ± 0.14a	104.09 ± 1.49c	34.06 ± 0.36a
	9	57.32 ± 0.55d	−8.64 ± 0.55c	32.06 ± 0.56a	105.09 ± 1.18bc	33.21 ± 0.40a
	12	56.61 ± 0.34d	−9.20 ± 0.34c	28.25 ± 1.42b	108.05 ± 0.24b	29.72 ± 1.45b
	15	48.98 ± 0.21e	−9.82 ± 0.21c	25.26 ± 2.87b	111.28 ± 1.71a	27.11 ± 2.75b
Type of cracker						
Gluten-free		59.09 ± 11.05b	−7.82 ± 1.74a	32.08 ± 3.94a	104.10 ± 4.61a	33.11 ± 3.41a
Wheat-based		62.59 ± 11.65a	−7.83 ± 1.95a	31.17 ± 3.66a	104.49 ± 4.91a	32.24 ± 3.14a

^1^Means followed by the same letter within a column are not significantly different (*p* > 0.05). ^2^*Polygonum cognatum* powder ratio

### Physical and Hardness Properties of Crackers Samples

Physical and textural properties are important indicators of structural quality and baking performance in crackers. These parameters reflect dough rheology, baking behavior, and textural attributes, particularly crispness and consumer acceptability [14]. Their importance is further increased in gluten-free and fiber-enriched systems, where the absence of gluten and the presence of plant-based ingredients significantly alter dough structure. The physical properties of crackers, including diameter, thickness, spread ratio, and hardness, are presented in Table 2. Diameter showed a slight increase with increasing PCP levels in both formulations, those increases found significant above 3% PCP levels in gluten-free crackers and 6% PCP in wheat-based crackers. This expansion may reflect dilution of the starch–protein or gluten matrix, altering dough handling and spread during baking. Thickness differed substantially between formulations: gluten-free crackers were thinner (from 1.56 to 1.84 mm) than regular crackers (3.36–3.80 mm), due to the absence of gluten and weaker dough structure. Increasing PCP levels above %6 significantly reduced thickness in gluten-free samples, while only non-significant slight changes were observed in regular crackers. Consequently, the spread ratio (D/T) was higher in gluten-free crackers (23.10–28.25) than in wheat-based ones (11.06–13.07), indicating a flatter structure. The increase in spread ratio with PCP incorporation can be explained by the disruption of the continuous matrix and the dilution of structure-forming components, which promotes dough flow during baking, as previously reported for fiber-enriched bakery products [13, 15].

**Table 2:** Physical properties of cracker samples

Type of cracker	PCP^2^ ratios (%)	Diameter (mm)	Thickness (mm)	Spread ratio (D/T)	Hardness (g)
	0	42.48 ± 0.31c	1.84 ± 0.06a	23.10 ± 0.88d	454.95 ± 28.64d
Gluten-free	3	42.94 ± 0.08bc	1.72 ± 0.03ab	25.01 ± 0.40c	534.83 ± 18.92d
	6	43.20 ± 0.00b	1.66 ± 0.08ab	26.05 ± 0.81bc	679.45 ± 20.14c
	9	43.36 ± 0.08ab	1.60 ± 0.14b	27.14 ± 0.02ab	757.91 ± 44.51c
	12	43.52 ± 0.11ab	1.58 ± 0.03b	27.56 ± 0.44ab	871.36 ± 37.50b
	15	43.96 ± 0.51a	1.56 ± 0.08b	28.25 ± 0.71a	997.74 ± 79.21a
Wheat-based	0	41.88 ± 0.03c	3.80 ± 0.57a	11.06 ± 0.05c	895.66 ± 30.33e
	3	42.06 ± 0.20c	3.78 ± 0.40a	11.24 ± 0.60c	1046.13 ± 35.88e
	6	42.42 ± 0.17bc	3.58 ± 0.45a	11.89 ± 0.12bc	1455.56 ± 41.80d
	9	42.58 ± 0.40bc	3.46 ± 0.37a	12.43 ± 1.16ab	2373.53 ± 67.13c
	12	42.86 ± 0.48ab	3.40 ± 0.14a	12.69 ± 0.10ab	3484.45 ± 41.79b
	15	43.58 ± 0.11a	3.36 ± 0.06a	13.07 ± 0.39a	4020.98 ± 97.61a
***Type of cracker***					
Gluten-free		43.24 ± 0.51a	1.66 ± 0.11b	26.19 ± 1.90a	716.04 ± 203.75b
Wheat-based		42.56 ± 0.61b	3.56 ± 0.19a	12.06 ± 0.81b	2212.72 ± 1310.10a

^1^Means followed by the same letter within a column are not significantly different (*p* > 0.05). ^2^*Polygonum cognatum* powder

Hardness exhibited the most pronounced changes among the physical parameters (Table 2). Gluten-free crackers showed a moderate increase in hardness (from 454.95 to 997.74 g), whereas regular crackers demonstrated a substantially greater increase (from 895.66 to 4020.98 g). On average, wheat-based crackers were more than three times harder than gluten-free samples. This behavior can be attributed to the strengthening effect of the gluten network and its interaction with dietary fiber and phenolic compounds present in PCP, resulting in a more rigid structure. Dietary fibers may compete with starch and gluten proteins for available water, limiting matrix plasticization and promoting the formation of a denser and firmer structure during baking [16]. Similar increases in hardness have been reported in bakery systems enriched with fiber-rich plant materials [13].

### Chemical Properties of Crackers Samples

The chemical composition of crackers was significantly influenced by both formulation type and the level of PCP addition (Table 3). Moisture content increased progressively with PCP incorporation, from 5.86 to 10.47% in gluten-free crackers and from 7.18 to 10.27% in regular crackers. This increase can be attributed to the high water-binding capacity of dietary fiber and hydrophilic constituents present in PCP, which enhance water retention within the product matrix [5]. Ash content, an indicator of mineral composition, increased markedly with PCP addition, reaching 2.72% in gluten-free and 2.88% in wheat-based crackers at 15% substitution compared to approximately 1.3% in its’ control samples. This trend reflects the naturally high mineral content of *P. cognatum*, consistent with previous findings [17, 18]. Protein content also increased significantly with PCP incorporation, from 5.13 to 8.34% in gluten-free crackers and from 10.93 to 13.52% in wheat-based crackers. While the higher baseline values in wheat based crackers are related to wheat protein content, the observed increase in both formulations can be attributed to the protein contribution of PCP and the concentration effect resulting from partial flour replacement. Fat content showed a moderate but significant increase (from 13.34 to 14.79% in gluten-free and 12.59 to 14.46% in wheat-based crackers). The most pronounced change was observed in total dietary fiber (TDF), which increased from 1.12 to 7.32% in gluten-free crackers and from 1.97 to 8.32% in regular crackers. This substantial increase confirms the potential of PCP as a fiber-rich functional ingredient and is consistent with previous studies reporting the nutritional potential of *Polygonum cognatum* and other fiber-rich plant materials used in cereal-based products [5, 18]. Dietary fibers derived from plant-based ingredients are known to improve water retention, modify product structure, and enhance the nutritional quality of bakery products [16]. From a nutritional perspective, increased dietary fiber intake has been associated with improved gastrointestinal health, enhanced satiety, modulation of postprandial glucose response, and a reduced risk of metabolic disorders such as obesity and type 2 diabetes [19].

**Table 3:** Chemical compositions of cracker samples^1^

Type of cracker	PCP^2^ ratios (%)	Moisture (%)	Ash (%)	Protein (%)	Fat (%)	TDF (%)	Phytic acid (mg/100 g)
	0	5.86 ± 0.18e	1.28 ± 0.01d	5.13 ± 0.11f	13.34 ± 0.01e	1.12 ± 0.09f	292.51 ± 6.03a
Gluten-free	3	7.53 ± 0.06d	1.58 ± 0.00c	5.74 ± 0.01e	13.75 ± 0.03d	2.30 ± 0.11e	264.65 ± 2.11b
	6	7.61 ± 0.01d	1.68 ± 0.06c	6.35 ± 0.02d	13.85 ± 0.05d	3.61 ± 0.08d	259.43 ± 7.41c
	9	7.91 ± 0.15c	2.14 ± 0.02b	7.03 ± 0.01c	14.11 ± 0.03c	4.82 ± 0.17c	216.77 ± 2.84d
	12	8.59 ± 0.03b	2.63 ± 0.00a	7.61 ± 0.01b	14.33 ± 0.01b	5.82 ± 0.22b	205.45 ± 5.02e
	15	10.47 ± 0.06a	2.72 ± 0.08a	8.34 ± 0.01a	14.79 ± 0.06a	7.32 ± 0.09a	177.60 ± 6.23f
Wheat-based	0	7.18 ± 0.06d	1.33 ± 0.09c	10.93 ± 0.06e	12.59 ± 0.00e	1.97 ± 0.06f	248.98 ± 3.19a
	3	8.37 ± 0.26c	1.58 ± 0.04c	11.47 ± 0.05d	12.94 ± 0.20d	3.24 ± 0.26e	240.28 ± 5.63b
	6	9.17 ± 0.03b	1.97 ± 0.09b	12.23 ± 0.18c	13.37 ± 0.05c	4.40 ± 0.12d	238.53 ± 17.20c
	9	10.05 ± 0.16a	2.14 ± 0.03b	12.95 ± 0.11b	13.79 ± 0.07b	5.65 ± 0.19c	229.83 ± 7.87d
	12	10.16 ± 0.08a	2.23 ± 0.00b	13.11 ± 0.07ab	13.89 ± 0.05b	6.93 ± 0.29b	198.49 ± 3.43e
	15	10.27 ± 0.28a	2.88 ± 0.10a	13.52 ± 0.29b	14.46 ± 0.05a	8.32 ± 0.36a	153.43 ± 8.46f
***Type of cracker***							
Gluten-free		7.99 ± 1.51b	2.01 ± 0.59a	6.70 ± 1.19b	14.03 ± 0.50a	4.17 ± 2.29b	236.07 ± 43.07a
Wheat-based		9.20 ± 1.23a	2.02 ± 0.54a	12.37 ± 1.01a	13.50 ± 0.68b	5.09 ± 1.37a	218.26 ± 36.24b

^1^Means followed by the same letter within a column are not significantly different (*p* > 0.05). Chemical properties are based on dry matter. Values are the average of triplicate measurements on the duplicate samples. ^2^*Polygonum cognatum* powder

Conversely, phytic acid content decreased significantly with increasing PCP levels, from 292.51 to 177.60 mg/100 g in gluten-free and from 248.98 to 153.43 mg/100 g in wheat-based crackers. This reduction may be attributed to the lower phytic acid content of PCP compared to cereal flours and the dilution of phytic acid-rich ingredients. From a nutritional perspective, this is advantageous, as phytic acid is known to chelate minerals and reduce their bioavailability. The reduction in phytic acid may improve the bioaccessibility of essential minerals such as Fe, Ca, and Zn in the final products [20].

### Chlorophyll, Antioxidant Capacity and Phenolic Composition of Crackers Samples

Phenolic compounds, antioxidant capacity, and chlorophyll are important dietary bioactives associated with several health-promoting effects. Therefore, enrichment of cereal-based products with phenolic-rich ingredients such as PCP may improve their functional and nutritional value. In this study, the incorporation of PCP significantly enhanced the bioactive compound profile and antioxidant capacity of both gluten-free and wheat-based crackers (Table 4). Total chlorophyll content increased consistently with increasing PCP levels, rising from 53.98 to 250.25 mg/kg in gluten-free crackers and from 48.69 to 212.36 mg/kg in wheat-based crackers. This increase reflects the high chlorophyll content of PCP and confirms its role as a natural source of plant pigments [18]. Antioxidant activity, evaluated using DPPH, FRAP, and CUPRAC assays, showed a substantial and dose-dependent increase with PCP incorporation. DPPH values increased from 175.19 to 5135.41 mg TE/kg in gluten-free crackers and from 36.04 to 4877.04 mg TE/kg in wheat-based crackers. Similar trends were observed for FRAP (from 1.76 to 23.47 µmol TE/g and from 1.13 to 22.49 µmol TE/g) and CUPRAC values (from 0.29 to 46.90 µmol TE/g and from 0.16 to 47.07 µmol TE/g). These increases are mainly attributed to the high phenolic content of PCP, as phenolic compounds are known to contribute significantly to antioxidant capacity in plant-based foods [21, 22]. Phenolic content, including free, bound, and total fractions, increased markedly with PCP addition. Total phenolic content rose from 3635.07 to 8300.74 mg GAE/kg in gluten-free crackers and from 4594.46 to 8782.70 mg GAE/kg in wheat-based crackers. In all samples, bound phenolics were the dominant fraction, which is typical for plant-based materials where phenolic compounds are associated with cell wall components [23]. Bound phenolics are particularly important from a nutritional perspective because they may reach the colon relatively intact and exert antioxidant effects through microbial fermentation processes [24]. Wheat-based crackers generally exhibited higher phenolic contents than gluten-free counterparts at equivalent PCP levels, which may be attributed to the contribution of wheat-derived phenolics [25]. The strong increase in antioxidant activity observed in all assays was consistent with the increase in total phenolic content, confirming the close relationship between phenolic compounds and antioxidant capacity in cereal-based systems enriched with plant materials [21].

**Table 4:** Chlorophyll, antioxidant activity (DPPH, FRAP and CUPRAC) and phenolic content (free, bound and total) of cracker samples^1^

Cracker type	PCP^2^ ratios (%)	Chlorophyll content			Antioxidant activity			Phenolic content		
		Chlorophyll-a (mg/kg)	Chlorophyll-b (mg/kg)	Total chlorophyll (mg/kg)	DPPH^3^ (mg TE/kg)	FRAP^4^ (µmol TE/g)	CUPRAC^5^ (µmol TE/g)	FPC^6^ (mg GAE/kg)	BPC^7^ (mg GAE/kg)	TPC^8^ (mg GAE/kg)
Gluten-free	0	16.50 ± 3.99f	34.74 ± 0.00e	53.98 ± 0.00f	175.19 ± 0.00f	1.76 ± 0.04f	0.29 ± 0.03f	1110.56 ± 90.85f	2524.51 ± 99.76f	3635.07 ± 8.92f
	3	48.45 ± 4.96e	48.90 ± 13.07de	97.22 ± 18.00e	1375.54 ± 16.64e	5.81 ± 0.12e	14.76 ± 0.45e	1666.88 ± 27.71e	2704.80 ± 28.34e	4371.68 ± 56.05e
	6	75.81 ± 6.18d	58.68 ± 2.73 cd	140.89 ± 8.90d	1853.89 ± 148.54d	8.68 ± 0.24d	21.48 ± 0.07d	2149.57 ± 2.39d	3401.79 ± 24.13d	5551.36 ± 107.62d
	9	87.92 ± 2.49c	65.25 ± 10.78bc	146.43 ± 13.25c	3020.67 ± 97.58c	10.50 ± 0.05c	28.47 ± 1.38c	2420.85 ± 80.50c	4050.46 ± 60.33c	6471.31 ± 20.16c
	12	110.31 ± 3.30b	74.61 ± 3.82ab	184.71 ± 7.11b	4086.01 ± 102.82b	17.04 ± 0.00b	39.17 ± 0.00b	2762.83 ± 18.97b	4684.44 ± 19.17b	7447.28 ± 38.15b
	15	155.65 ± 1.25a	94.88 ± 2.35a	250.25 ± 0.00a	5135.41 ± 136.77a	23.47 ± 0.46a	46.90 ± 1.69a	3106.34 ± 0.00a	5032.95 ± 35.28a	8300.74 ± 60.29a
Wheat-based	0	17.43 ± 6.16f	31.33 ± 1.08c	48.69 ± 17.21e	36.04 ± 0.00f	1.13 ± 0.03e	0.16 ± 0.00f	1503.68 ± 3.21f	3090.79 ± 262.10e	4594.46 ± 129.44f
	3	44.42 ± 6.66e	40.92 ± 9.19c	85.24 ± 2.51d	1161.10 ± 64.90e	4.12 ± 1.08d	11.64 ± 0.06e	2166.86 ± 94.90e	3569.19 ± 106.45d	5736.05 ± 28.59e
	6	62.92 ± 4.94d	57.33 ± 10.30bc	120.10 ± 5.35d	1707.88 ± 173.23d	8.49 ± 1.06c	18.11 ± 0.36d	2371.00 ± 1.82d	4125.18 ± 2.13c	6496.18 ± 3.95d
	9	78.71 ± 5.82c	68.48 ± 17.62ab	136.03 ± 11.77c	2772.09 ± 18.50c	9.92 ± 0.53c	27.98 ± 1.45c	2585.06 ± 46.80c	4693.13 ± 100.79b	7278.19 ± 154.79c
	12	109.59 ± 8.55b	79.18 ± 17.54a	188.55 ± 26.05b	3857.85 ± 106.56b	15.66 ± 0.23b	37.77 ± 4.17b	2987.34 ± 99.74b	5282.71 ± 38.79a	8120.72 ± 150.23b
	15	131.38 ± 3.55a	81.22 ± 10.67a	212.36 ± 14.20a	4877.04 ± 44.80a	22.49 ± 1.34a	47.07 ± 0.98a	3347.25 ± 50.63a	5474.79 ± 65.64a	8782.70 ± 40.63a
***Cracker type***		**Chlorophyll-a (mg/kg)**	**Chlorophyll-b (mg/kg)**	**Total chlorophyll (mg/kg)**	**DPPH** ^**3**^ **(mg TE/kg)**	**FRAP** ^**4**^ **(µmol TE/g)**	**CUPRAC** ^**5**^ **(µmol TE/g)**	**FPC** ^**6**^ **(mg GAE/kg)**	**BPC** ^**7**^ **(mg GAE/kg)**	**TPC** ^**8**^ **(mg GAE/kg)**
Gluten-free		236.07 ± 43.07a	82.44 ± 48.40a	62.84 ± 20.84a	2607.79 ± 1830.24a	11.21 ± 7.87a	23.79 ± 17.29a	2202.84 ± 729.47b	3733.16 ± 1031.82b	5962.91 ± 1791.51b
Wheat-based		218.26 ± 36.24b	74.07 ± 41.90b	57.91 ± 19.96b	2402.00 ± 1788.95b	10.30 ± 7.79b	25.18 ± 16.85b	2493.53 ± 645.39a	4372.63 ± 948.22a	6834.72 ± 1548.26a

^1^Means followed by the same letter within a column are not significantly different (*p* > 0.05). Chemical properties are based on dry matter. Values are the average of triplicate measurements on the duplicate samples. ^2^*Polygonum cognatum* powder. ^3^2,2-diphenyl-1-picrylhydrazyl radical scavenging activity (TE: Trolox equivalent). ^4^Ferric reducing antioxidant power. ^5^Cupric ion reducing antioxidant capacity. ^6^Free phenolic content (GAE: gallic acid equivalent). ^7^Bound phenolic content. ^8^Total phenolic content

### Glycemic Index (eGI) of Crackers

The glycemic index reflects the postprandial blood glucose response to carbohydrate-containing foods and is a key indicator of their nutritional quality [26]. The consumption of low-GI foods is associated with improved glycemic control, reduced insulin demand, and a lower risk of chronic metabolic diseases such as type 2 diabetes, obesity, and cardiovascular disorders [27]. In addition, low-GI diets may enhance satiety and support weight management. Therefore, reducing the GI of cereal-based products is an effective strategy to improve their nutritional and functional value. The eGI (estimated glycemic index) values of the cracker samples decreased significantly with increasing PCP incorporation in both gluten-free and wheat-based formulations (Figure S2). In gluten-free crackers, eGI decreased from 96.57 in the control sample to 60.12 at 15% PCP, while in wheat-based crackers, it decreased from 92.45 to 57.98, indicating a clear dose-dependent reduction. This reduction in eGI can be mainly attributed to the high dietary fiber content of PCP (42.84%, data not shown), which plays a crucial role in reducing starch digestibility. Dietary fiber is known to limit enzymatic access to starch by forming a physical barrier and increasing the viscosity of the digestion medium, thereby slowing glucose release [5, 7]. In addition to dietary fiber, the high phenolic content of PCP may also contribute to the reduction in eGI. Phenolic compounds have been reported to interact with digestive enzymes and reduce starch hydrolysis, leading to a slower release of glucose [21, 23]. The strong increase observed in total phenolic content and antioxidant activity in the present study supports this mechanism. Another important factor is the structural modification of the food matrix. The incorporation of PCP likely disrupted the starch–protein network and reduced starch availability for enzymatic hydrolysis. In addition, phenolic compounds may form complexes with starch and digestive enzymes, further slowing starch hydrolysis kinetics [28]. Such structural changes have been reported to influence starch digestion kinetics in cereal-based products [7]. Gluten-free crackers consistently exhibited slightly higher eGI values than regular crackers at all substitution levels. This can be explained by the higher digestibility of starch in gluten-free formulations, which are mainly composed of rice flour and corn starch. These ingredients typically produce higher glycemic responses due to their more accessible starch structure, whereas wheat-based systems may form a more compact matrix that can partially limit enzymatic digestion. At higher PCP levels (≥ 12%), both cracker types reached substantially lower eGI values, indicating a significant nutritional improvement. These findings suggest that PCP incorporation is an effective strategy for reducing the glycemic impact of cereal-based products through the combined effects of dietary fiber enrichment, phenolic compounds, and structural modifications of the food matrix.

Pearson correlation analysis revealed strong relationships between bioactive compounds, antioxidant capacity, and glycemic response (Figure S3). A highly significant negative correlation was observed between eGI and total phenolic content (*r* = − 0.96), as well as free (*r* = − 0.94) and bound phenolics (*r* = − 0.95), indicating that phenolic compounds played a key role in reducing starch digestibility. Similarly, eGI showed strong negative correlations with antioxidant activity parameters, including DPPH (*r* = − 0.94), FRAP (*r* = − 0.93), and CUPRAC (*r* = − 0.93), suggesting that bioactive compounds contributed to the modulation of glycemic response. Strong positive correlations were observed between total phenolic content and antioxidant activity (*r* = 0.91–0.94), confirming that phenolic compounds were the main contributors to antioxidant capacity. In addition, hardness exhibited a moderate to strong positive correlation with phenolic fractions (*r* = 0.68–0.76) and a strong negative correlation with eGI (*r* = − 0.76), indicating that structural changes in the product matrix may also influence starch hydrolysis. These results demonstrate that both compositional (phenolic compounds) and structural (texture) factors contribute synergistically to the reduction of glycemic index in PCP-enriched crackers.

### Mineral Composition of Crackers Samples

The incorporation of PCP markedly enhanced the mineral composition (Ca, Fe, Mg, K, P and Zn) of both gluten-free and wheat-based crackers (Table 5). Ca showed the most pronounced increase, rising from 4.78 to 125.55 mg/100 g in gluten-free and from 18.21 to 135.20 mg/100 g in wheat-based crackers, which reflects the high Ca content of PCP (479.97 mg/100 g data not shown). This pronounced increase is consistent with the naturally high Ca concentration of *P. cognatum* [18, 29]. The nutritional relevance of this finding is notable, as Ca deficiency remains prevalent in cereal-based diets and adequate intake is essential for bone development and maintenance [30]. Fe also increased significantly (*p* < 0.05), while Mg and K contents rose steadily, reaching 81.56 and 399.87 mg/100 g in gluten-free crackers and 92.49 and 423.56 mg/100 g in wheat-based crackers, respectively. Similarly, P content increased notably with PCP addition. These increases reflect the mineral-rich composition of PCP, particularly in terms of Ca, Fe, Mg, K, and P, and highlight its nutritional potential. These improvements are of dietary importance, given that Fe deficiency is one of the most widespread micronutrient deficiencies globally. The fortification of cereal products with iron-rich leafy plants has been proposed as an effective strategy for enhancing iron intake [31]. In contrast, Mn showed only moderate increases, whereas Cu and Zn remained largely unchanged, indicating that PCP mainly contributes to macro-minerals rather than trace elements. Wheat-based crackers generally exhibited higher Ca, Fe, Mg, K, and Mn values than gluten-free ones, likely due to the additional contribution of wheat flour minerals, while Ca and P contents became comparable at higher substitution levels, suggesting that PCP was the dominant mineral source at higher incorporation ratios.

**Table 5:** Mineral contents (mg/100 g dry matter) of cracker samples^1^

Type of cracker	PCP^2^ ratios (%)	Ca	Fe	Cu	Mg	K	Mn	*P*	Zn
Gluten-free	0	4.78 ± 0.63f	0.72 ± 0.41f	0.14 ± 0.01a	30.41 ± 0.18f	113.75 ± 0.70f	1.12 ± 0.08b	345.18 ± 1.98f	1.09 ± 0.11a
	3	28.30 ± 1.71e	1.65 ± 0.40e	0.14 ± 0.00a	41.32 ± 1.78e	173.12 ± 2.02e	1.15 ± 0.04ab	364.25 ± 1.74e	1.11 ± 0.00a
	6	51.62 ± 0.39d	2.62 ± 0.45d	0.13 ± 0.04a	51.74 ± 1.34d	233.83 ± 1.06d	1.18 ± 0.01ab	382.52 ± 2.06d	1.15 ± 0.00a
	9	76.76 ± 0.70c	3.64 ± 0.24c	0.14 ± 0.04a	62.47 ± 1.50c	285.04 ± 2.02c	1.25 ± 0.08ab	399.46 ± 2.35c	1.13 ± 0.06a
	12	102.06 ± 1.48b	4.62 ± 0.18b	0.15 ± 0.06a	71.47 ± 0.76b	351.71 ± 1.68b	1.27 ± 0.03ab	413.29 ± 2.21b	1.19 ± 0.03a
	15	125.55 ± 0.42a	5.72 ± 0.27a	0.15 ± 0.08a	81.56 ± 1.39a	399.87 ± 2.42a	1.31 ± 0.03a	432.54 ± 2.01a	1.21 ± 0.06a
Wheat-based	0	18.21 ± 0.17f	0.93 ± 0.11f	0.14 ± 0.01a	36.41 ± 0.58f	130.43 ± 1.17f	1.24 ± 0.06c	334.55 ± 1.00f	1.05 ± 0.06a
	3	41.54 ± 0.79e	1.88 ± 0.07e	0.15 ± 0.01a	48.22 ± 1.03e	200.36 ± 2.96e	1.29 ± 0.01c	352.26 ± 3.20e	1.08 ± 0.04a
	6	65.33 ± 0.46d	2.90 ± 0.06d	0.15 ± 0.01a	59.96 ± 1.32d	250.46 ± 1.58d	1.33 ± 0.03bc	370.51 ± 2.87d	1.11 ± 0.06a
	9	88.75 ± 1.05c	3.80 ± 0.02c	0.16 ± 0.01a	70.30 ± 1.34c	318.91 ± 1.35c	1.37 ± 0.06abc	381.23 ± 3.27c	1.13 ± 0.06a
	12	110.63 ± 2.66b	4.70 ± 0.07b	0.17 ± 0.03a	81.24 ± 2.26b	379.65 ± 2.35b	1.43 ± 0.03ab	392.14 ± 4.71b	1.17 ± 0.06a
	15	135.20 ± 2.32a	5.80 ± 0.16a	0.18 ± 0.04a	92.49 ± 1.48a	423.56 ± 2.05a	1.48 ± 0.07a	409.85 ± 3.80a	1.22 ± 0.04a
**Type of cracker**									
Gluten-free		64.84 ± 43.35b	3.16 ± 1.80b	0.14 ± 0.04a	56.50 ± 18.22b	259.55 ± 102.89b	1.21 ± 0.08b	389.54 ± 30.69a	1.15 ± 0.06a
Wheat-based		76.61 ± 41.59a	3.34 ± 1.72a	0.16 ± 0.02a	64.77 ± 19.90a	283.90 ± 105.80a	1.36 ± 0.09a	373.42 ± 26.07b	1.13 ± 0.07a

^1^Means followed by the same letter within a column are not significantly different (*p* > 0.05). Values are the average of triplicate measurements on the duplicate samples. ^2^*Polygonum cognatum* powder

###  Sensory Properties of Crackers Samples

The sensory properties of the cracker samples enriched with PCP are presented in Figure S4 for gluten-free and wheat-based formulations. For wheat-based crackers, color, appearance, taste, odor, crispness, and overall acceptability scores were highest in the control and in samples containing 3–6% PCP. At these levels, PCP incorporation contributed positively to visual attributes, likely due to the development of a desirable greenish tone without excessive darkening. However, color and appearance scores decreased progressively at higher substitution levels, with the lowest values observed at 15% PCP, reflecting the negative impact of excessive darkening. Taste and odor scores also declined beyond 9% PCP, which may be attributed to the development of more intense plant-derived flavors. Crispness values were maintained up to 6% PCP but decreased significantly at higher substitution levels, particularly at 12–15%, indicating that excessive fiber incorporation negatively affected product texture and friability. Consequently, overall acceptability scores were significantly higher in the control and 3–6% PCP samples compared to higher substitution levels. A similar trend was observed in gluten-free crackers. Low to moderate PCP additions (3–6%) resulted in sensory scores comparable to the control for most attributes, whereas higher levels (12–15%) led to significant decreases across all sensory parameters. The reduction was particularly pronounced for crispness and overall acceptability. These findings are consistent with previous studies reporting that incorporation of plant powders improves functional properties but may adversely affect sensory quality at high substitution levels [32].

## Conclusion

This study demonstrated that incorporation of PCP significantly improved the nutritional, bioactive, and functional properties of both gluten-free and wheat-based crackers. PCP enrichment increased protein, dietary fiber, phenolic content, antioxidant capacity, and some essential minerals, while reducing phytic acid content. Moreover, PCP effectively lowered the estimated glycemic index, indicating its potential to produce healthier cereal-based products with reduced glycemic response. However, high substitution levels (≥ 12%) negatively affected sensory attributes such as color, taste, and crispness, whereas moderate incorporation levels (3–6%) provided the best balance between functional enhancement and consumer acceptability. Overall, PCP can be considered a promising natural ingredient for the development of functional bakery products. Future studies should focus on shelf-life stability, consumer acceptance in larger populations, and industrial-scale applicability.

## Supplementary Information

Below is the link to the electronic supplementary material.

Supplementary Material 1 (DOCX 756 KB)

## Data Availability

The datasets generated and/or analyzed during the current study are available from the corresponding author on reasonable request.
