# Supplementary material for: Polygonum cognatum Powder as a Functional Ingredient: Improving Nutritional Quality and Reducing Glycemic Index of Gluten-free and Wheat-based Crackers
Source: Plant Foods Hum Nutr. 2026 May 21;81(2):66. doi: 10.1007/s11130-026-01518-9 (PMC13194194; doi:10.1007/s11130-026-01518-9)
Supplement: Supplementary file 1 — Supplementary Material 1 (DOCX 756 KB) [file 11130_2026_1518_MOESM1_ESM.docx]

**Supplementary Materials**

***Polygonum cognatum* Powder as a Functional Ingredient: Improving Nutritional Quality and Reducing Glycemic Index of Gluten-Free and Wheat-Based Crackers**

**Tekmile Cankurtaran-Kömürcü^a*^ Nermin Bilgiçli^a^**

*^a^Department of Food Engineering, Engineering Faculty, Necmettin Erbakan University, Köyceğiz Campus, Konya, 42050, Türkiye.*

*corresponding-author: tekmilecankurtaran@gmail.com

**Materials and Methods**

**Plant samples**

*Polygonum cognatum* samples were harvested in May 2023 from the Central Anatolia region of Türkiye (Başınayayla, Yozgat) and transported to the laboratory under refrigerated conditions. The samples were stored at +4 °C and processed within 24 h. Prior to drying, foreign materials and damaged plant parts were manually removed, and the samples were washed with distilled water to eliminate surface impurities.

Fresh samples were dried in a laboratory-scale vacuum oven (JSVO-60T, JS Research Inc., Seoul, Korea) at 40 °C for 18 h until the moisture content decreased below 10% (wet basis), ensuring product stability and minimizing microbial deterioration. The dried samples were ground using a laboratory mill and sieved through a 500 µm mesh to obtain a homogeneous powder. The resulting *P. cognatum* powder (PCP) was packed in airtight containers and stored at −18 °C until further analyses and cracker production.

For cracker preparation (both regular and gluten-free), refined wheat flour (*Triticum compactum* Host.; Ova, Konya, Türkiye), rice flour (Dr. Oetker, İzmir, Türkiye), corn starch (Dr. Oetker, İzmir, Türkiye), shortening (Dr. Oetker, İzmir, Türkiye), salt (Avrasya, Konya, Türkiye), powdered sugar (Dr. Oetker, İzmir, Türkiye), baking powder (Dr. Oetker, İzmir, Turkey), and fresh compressed baker’s yeast (Pakmaya, İzmir, Türkiye) were obtained from a local market in Konya (Türkiye). The protease enzyme was supplied by Vatan Enzyme (İstanbul, Türkiye) and was used to partially hydrolyze protein structures in the dough, thereby reducing dough elasticity, improving machinability, and contributing to the development of a desirable crisp texture in wheat-based.

**Preparation of crackers**

Cracker samples were prepared with slight modifications to the method described by Kömürcü [1]. Two formulations were developed: regular wheat-based (gluten-containing) and gluten-free. The control wheat-based cracker dough consisted of refined wheat flour (100 g), shortening (20 g), salt (1.6 g), powdered sugar (1.5 g), baking powder (1.5 g), baker’s yeast (0.2 g), protease (0.01 g), and water (16 mL). For gluten-free crackers, wheat flour was replaced with a 1:1 mixture of corn starch and rice flour (50 g each), while other ingredients remained unchanged. All ingredients were mixed using a mixer (Hobart N50, Canada Inc., North York, Ontario, Canada) until a homogeneous dough was obtained. The dough was fermented at ambient temperature for 20 min, sheeted to 1 mm thickness, and cut into discs (50 mm diameter). Baking was carried out in a household oven (Vestel SF8401, Manisa, Türkiye) at 180 °C for 11 min.

PCP was incorporated at levels of 0, 3, 6, 9, 12, and 15% (w/w flour basis). Detailed formulations and images of cracker samples are provided in Supplementary Materials (**Table S1 and Figure S1**).

**Table S1.** Formulation of cracker samples

| **Ingredients**  **(g)** | **Wheat-based** | | | | | |  | **Gluten-free** | | | | | |
| --- | --- | --- | --- | --- | --- | --- | --- | --- | --- | --- | --- | --- | --- |
|  | **0%** | **3%** | **6%** | **9%** | **12%** | **15%** |  | **0%** | **3%** | **6%** | **9%** | **12%** | **15%** |
| **Wheat flour** | 100 | 97 | 94 | 91 | 88 | 85 |  | - | - | - | - | - | - |
| **Corn starch** | - | - | - | - | - | - |  | 50 | 48.5 | 47 | 45.5 | 44 | 42.5 |
| **Rice flour** | - | - | - | - | - | - |  | 50 | 48.5 | 47 | 45.5 | 44 | 42.5 |
| **PCP** | 0 | 3 | 6 | 9 | 12 | 15 |  | 0 | 3 | 6 | 9 | 12 | 15 |
| **Shortening** | 20 | 20 | 20 | 20 | 20 | 20 |  | 20 | 20 | 20 | 20 | 20 | 20 |
| **Salt** | 1.6 | 1.6 | 1.6 | 1.6 | 1.6 | 1.6 |  | 1.6 | 1.6 | 1.6 | 1.6 | 1.6 | 1.6 |
| **Sugar** | 1.5 | 1.5 | 1.5 | 1.5 | 1.5 | 1.5 |  | 1.5 | 1.5 | 1.5 | 1.5 | 1.5 | 1.5 |
| **Baking powder** | 1.5 | 1.5 | 1.5 | 1.5 | 1.5 | 1.5 |  | 1.5 | 1.5 | 1.5 | 1.5 | 1.5 | 1.5 |
| **Baker’s yeast** | 0.2 | 0.2 | 0.2 | 0.2 | 0.2 | 0.2 |  | 0.2 | 0.2 | 0.2 | 0.2 | 0.2 | 0.2 |
| **Protease** | 0.01 | 0.01 | 0.01 | 0.01 | 0.01 | 0.01 |  | - | - | - | - | - | - |

PCP*: Polygonum cognatum* powder.


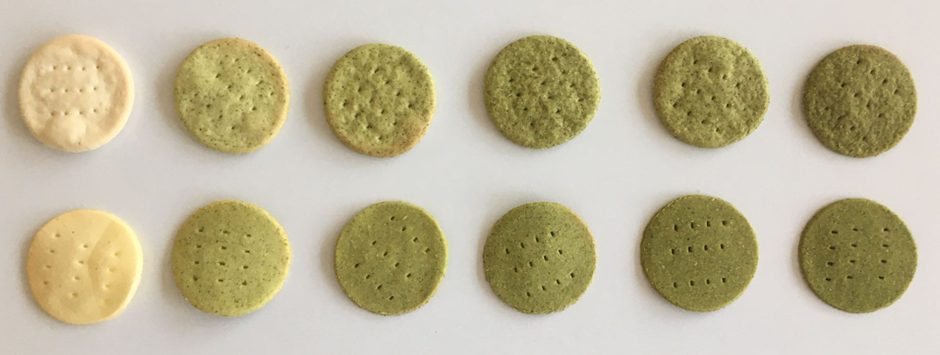


A

**Control**

B

**15%**

**12%**

**9%**

**3%**

**6%**

**Figure S1.** Wheat-based (A) and Gluten-free (B) cracker samples

**Color measurement**

The color values of the cracker samples were determined using a Minolta CR-400 (Hunter Lab Chroma Meter, Osaka, Japan). Measurements were taken from five different points on the surface of each cracker. The L* (brightness), a* (red–green), and b* (yellow–blue) values were recorded, and saturation index ((a²+b²)¹/²) and Hue angle (a*>0 and b*>0, if arctan [b*/a*]; a*<0 and b*>0, arctan [b*/a*] + 180°) were calculated. A white tile with color values L* = 98.45, a* = –0.10, b* = –0.13 was used as a reference.

**Physical properties**

The physical attributes of the final products, including diameter, thickness, spread ratio, and textural characteristics, were examined. Diameter and thickness were determined using a digital caliper (Mitutoyo, Tokyo, Japan) on five individual samples, following the AACC 10-54 standard protocol [2], and results were expressed in millimeters. The spread ratio was calculated by dividing the diameter of the cracker by its thickness.

Textural properties, specifically hardness and fracturability, were assessed using a TA-XT Plus texture analyzer (Stable Micro Systems, Surrey, UK) fitted with a 5 kg load cell, applying a three-point bending (HDP/3PB) method. The test was conducted under the following conditions: pre-test speed of 1.0 mm/s, test speed of 1.0 mm/s, and post-test speed of 10.0 mm/s. For each formulation, five measurements were performed and the analysis was repeated twice.

**Determination of chemical composition**

Moisture, ash, protein and fat contents of samples were determined using the standard AACC [3] methods (44–19, 08–01, 46–12, and 30–25, respectively). Total dietary fiber (TDF) of cracker samples was quantified by the enzymatic gravimetric procedure described in the AOAC [4] guidelines.

Phytic acid content of the cracker samples was determined according to the method of Haug and Lantzsch [5]. Samples were extracted with 0.2 N HCl in a shaking water bath at 180 rpm for 2 hours at room conditions. After the obtained extract was treated with iron III solutions, the amount of iron remaining in the serum part was determined by the spectrophotometric method.

**Determination of chlorophyll content**

Chlorophyll content was determined by extracting 0.1 g of sample with 80% acetone and measuring absorbance at 663 and 645 nm using a spectrophotometer (Hitachi-U1800, Japan). Chlorophyll a, b, and total chlorophyll were calculated according to Ayadi et al. [6].

**Determination of free, bound and total phenolic content**

Free and bound phenolic compounds were extracted according to the method reported by Vitali et al. [7], with minor modifications. For the extraction of free phenolics, 1 g of powdered sample was mixed with 10 mL of methanol:water solution (80:20, v/v) containing 1% HCl. The mixture was placed in a shaking water bath and continuously agitated at room temperature (24 ± 1 °C) for 2 h. After extraction, the samples were centrifuged at 1058 ×g, and the supernatants were separated and stored at −20 °C until analysis. For bound phenolics, the remaining solid residue obtained after free phenolic extraction was treated with 20 mL of a methanol–sulfuric acid solution (10:1, v/v). The mixture was incubated in a shaking water bath at 85 °C for 20 h to facilitate the release of bound phenolic compounds. After cooling to room temperature, the samples were centrifuged at 1058 ×g, and the supernatants were collected and stored at −20 °C for further analysis.

The determination of free and bound phenolic contents was carried out using the Folin–Ciocalteu colorimetric method described by Naczk and Shahidi [8]. Absorbance was measured at 760 nm using a UV–Vis spectrophotometer (Hitachi-U1800, Japan). A calibration curve was prepared using gallic acid as the standard, and the results were expressed as gallic acid equivalents (GAE). Total phenolic content was calculated as the sum of free and bound phenolic fractions.

**Determination of antioxidant activity**

For the determination of antioxidant activity, sample extracts were prepared using an 80% methanol:water mixture (80:20, v/v), following the same extraction procedure applied in the total phenolic content (TPC) analysis. Antioxidant capacity was evaluated using three complementary assays. The radical scavenging activity was determined using the DPPH (2,2-diphenyl-1-picrylhydrazyl) method according to Beta et al. [9], and the results were expressed as mg Trolox equivalents per kg sample. The ferric reducing antioxidant power (FRAP) assay was performed based on the procedure described by Gao et al. [10], with results reported as μmol Trolox equivalents per g sample. In addition, the cupric ion reducing antioxidant capacity (CUPRAC) method was applied following Apak et al. [11], and the antioxidant capacity was expressed as μmol Trolox equivalents per g sample.

**Glycemic index**

In vitro starch digestion was carried out following the procedure described by Goñi et al. [12]. Initially, 50 mg of sample was accurately weighed and homogenized with 5 mL of distilled water. To mimic gastric conditions, 10 mL of 0.1 M HCl–KCl buffer (pH 1.5) and 0.2 mL of pepsin solution were added, and the mixture was incubated in a shaking water bath at 40 °C for 1 h. After the gastric phase, the intestinal phase was simulated by adding 10 mL of 0.1 M tris–maleate buffer (pH 6.9), and the total volume was adjusted to 25 mL. Subsequently, 5 mL of α-amylase solution (2.6 U) was introduced, and the samples were incubated at 37 °C for 3 h. During the enzymatic hydrolysis process, aliquots were withdrawn at 0, 20, 30, 60, 90, 120, and 180 min, and enzyme activity was immediately terminated by boiling. To ensure complete conversion of dextrins into glucose, each aliquot was treated with 3 mL of 0.4 M sodium acetate buffer (pH 4.75) and 60 µL of amyloglucosidase solution (Megazyme, diluted 100-fold), followed by incubation at 60 °C for 45 min. Glucose content was quantified using the GOPOD assay kit (Megazyme, Ireland). For this purpose, 30 µL of hydrolysate was mixed with 900 µL of GOPOD reagent and incubated at 50 °C for 20 min. Absorbance was measured at 510 nm using a UV–Vis spectrophotometer (Hitachi-U1800, Japan). Glucose concentrations were determined from a standard calibration curve, and starch hydrolysis curves were constructed accordingly. Fresh white bread was used as the reference sample. The amount of released glucose was converted into starch by applying a conversion factor of 0.9, and the extent of starch digestion was expressed as the percentage of total starch hydrolyzed at each sampling time (0, 20, 30, 60, 90, 120, and 180 min). A nonlinear model was applied to describe starch hydrolysis kinetics using a first-order equation:

$$C_{t}=C_{\infty}\left( 1 - e^{-kt} \right)$$

where:
$C_{t}$= starch hydrolyzed at time t, $C_{\infty}$= equilibrium starch hydrolysis (%), $k$= kinetic constant, $t$= time (min)

The area under the hydrolysis curve (AUC) from 0 to 180 min was calculated from the fitted model.

The hydrolysis index (HI) was calculated as:

$$HI=\frac{AUC_{sample}}{AUC_{reference}}\times100$$

White bread was used as the reference food, and its AUC value was considered as 100.

The estimated glycemic index (eGI) was calculated using the equation proposed by Goñi et al. [12]:

$$eGI=39.71+0.549\times HI$$

**Determination of mineral analysis**

Dried samples (0.3 g) were digested with a mixture of HNO₃ and H₂SO₄ (7:1 mL) using a microwave digestion system (Mars 5, CEM, USA). The mineral concentrations of Ca, Fe, K, Mg, P and Zn were then determined with ICP-MS (Agilent 7900, Germany) [13].

**Sensory evaluation**

Sensory evaluation was conducted with 30 semi-trained adult panelists (15 females, 15 males; 20–40 years old) recruited from the university staff and students. Participants were regular consumers of cereal-based snacks and reported no allergies to the ingredients used. The study was carried out in accordance with institutional ethical guidelines, and informed consent was obtained from all panelists. Wheat-based and gluten-free crackers containing PCP were freshly prepared and evaluated under controlled laboratory conditions (individual booths, neutral lighting, serving at room temperature). Samples were coded with three-digit random numbers and presented monadically in randomized order. Panelists rated color, aroma, taste, crispness, and overall acceptability using a 7-point hedonic scale (1 = dislike extremely, 7 = like extremely). Water was provided for palate cleansing between samples.

**Statistical analysis**

All analyses were conducted in triplicate, and data were expressed as mean ± standard deviation. Statistical differences among samples were determined using SPSS 22.0 software (SPSS Inc., Chicago, IL, USA). Duncan’s multiple range test was applied at a significance level of p < 0.05 to compare means.

**Figure S2.** Estimated glycemic index (eGI) values of gluten-free and wheat-based cracker samples enriched with different levels of *Polygonum cognatum* powder (0–15%). Different letters above the bars indicate statistically significant differences (p < 0.05).

**Figure S3.** Pearson correlation coefficients (r) between antioxidant activity (DPPH, FRAP, and CUPRAC), phenolic content (free, bound, and total), estimated glycemic index (eGI), and hardness (g) of cracker samples. Strong positive correlations were observed among phenolic content and antioxidant activity parameters, whereas eGI showed strong negative correlations with these variables.

|  |
| --- |
|  |

**Figure S4.** Sensory analysis results of gluten-free and wheat-based cracker samples with different levels of Polygonum cognatum powder incorporation.

**References**

1. Kömürcü TC (2023) Effect of hazelnut skin addition on quality characteristics of functional crackers. KSU J Agric Nat 26:1368–1376. https://doi.org/10.18016/ksutarimdoga.vi.1222490

2. AACC (2010) Approved methods of analysis, 11th edn. AACC International, St. Paul

3. AACC (2000) Approved methods of analysis, 10th edn. American Association of Cereal Chemists, St. Paul

4. AOAC (2012) Official methods of analysis, 19th edn. Association of Official Analytical Chemists, Gaithersburg

5. Haug W, Lantzsch HJ (1983) Sensitive method for the rapid determination of phytate in cereals and cereal products. J Sci Food Agric 34:1423–1426. https://doi.org/10.1002/jsfa.2740341217

6. Ayadi MA, Grati-Kamoun N, Attia H (2009) Physico-chemical change and heat stability of extra virgin olive oils flavoured by selected Tunisian aromatic plants. Food Chem Toxicol 47:2613–2619. https://doi.org/10.1016/j.fct.2009.07.024

7. Vitali D, Dragojević IV, Šebečić B (2009) Effects of incorporation of integral raw materials and dietary fibre on selected properties of biscuits. Food Chem 114:1462–1469. <https://doi.org/10.1016/j.foodchem.2008.11.032>

8. Naczk M, Shahidi F (2004) Extraction and analysis of phenolics in food. J Chromatogr A 1054:95–111. https://doi.org/10.1016/j.chroma.2004.08.059

9. Beta T, Nam S, Dexter JE, Sapirstein HD (2005) Phenolic content and antioxidant activity of wheat fractions. Cereal Chem 82:390–393. https://doi.org/10.1094/CC-82-0390

10. Gao X, Ohlander M, Jeppsson N et al (2000) Changes in antioxidant effects and their relationship to phytonutrients in fruits of sea buckthorn during maturation. J Agric Food Chem 48:1485–1490. https://doi.org/10.1021/jf991072g

11. Apak R, Güçlü K, Özyürek M, Çelik SE (2008) Mechanism of antioxidant capacity assays and the CUPRAC assay. Microchim Acta 160:413–419. <https://doi.org/10.1007/s00604-007-0777-0>

12. Goñi I, García-Alonso A, Saura-Calixto F (1997) A starch hydrolysis procedure to estimate glycemic index. Nutr Res 17:427–437. <https://doi.org/10.1016/S0271-5317(97)00010-9>

13. Skujins S (1998) Handbook for ICP-AES (Varian-Vista): A short guide to Vista operation. Department of Agronomy, Iowa State University, Ames.
